# Exosomes and their roles in the chemoresistance of pancreatic cancer

**DOI:** 10.1002/cam4.4830

**Published:** 2022-05-19

**Authors:** Yubin Pan, Honglin Tang, Qijun Li, Guangpeng Chen, Da Li

**Affiliations:** ^1^ Department of Medical Oncology, Sir Run Run Shaw Hospital Zhejiang University School of Medicine Hangzhou China

**Keywords:** apoptosis, cancer‐associated fibroblasts, chemotherapy, pancreatic cancer

## Abstract

Pancreatic cancer (PC) remains one of the most lethal human malignancies worldwide. Due to the insidious onset and the rapid progression, most patients with PC are diagnosed at an advanced stage rendering them inoperable. Despite the development of multiple promising chemotherapeutic agents as recommended first‐line treatment for PC, the therapeutic efficacy is largely limited by unwanted drug resistance. Recent studies have identified exosomes as essential mediators of intercellular communications during the occurrence of drug resistance. Understanding the underlying molecular mechanisms and complex signaling pathways of exosome‐mediated drug resistance will contribute to the improvement of the design of new oncologic therapy regimens. This review focuses on the intrinsic connections between the chemoresistance of PC cells and exosomes in the tumor microenvironment (TME).

## Graphical abstract

Despite the development of multiple promising chemotherapeutic agents as recommended first‐line treatment for PC, the therapeutic efficacy is largely limited by unwanted drug resistance.Exploring the relation between exosomes and chemoresistance may be conducive to understanding molecular mechanisms and taking effective measures to reduce the development of drug resistance.

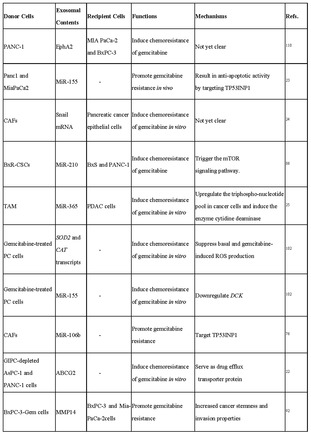

## 1 INTRODUCTION

Pancreatic cancer (PC) is an aggressive malignant tumor with a five‐year relative survival rate of less than 10%.^1^ For decades, surgery has been recommended as the suitable treatment for early PC, however, a large proportion of patients are diagnosed at an advanced stage rendering them inoperable.^2^, ^3^ Moreover, the US Food and Drug Administration (FDA)‐approved immunotherapies are almost completely inactive against PC except for the <1% of patients with microsatellite instability‐high (MSI‐H) tumors.^4^ Consequently, chemotherapy remains the mainstay of treatment of advanced‐stage PC.

**FIGURE 1:**
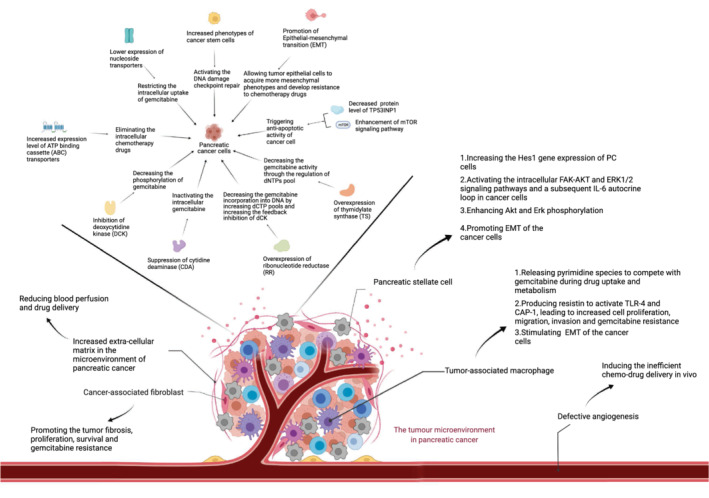
The principal mechanisms of gemcitabine resistance in pancreatic cancer

Currently, the first‐line chemotherapy regimens for locally advanced and metastatic PC are mainly limited to FOLFIRINOX, modified FOLFIRINOX, and gemcitabine‐based multidrug combination.^5^ Due to the interaction among PC cells, cancer stem cells, and the tumor microenvironment (TME), the development of multifactorial chemoresistance leads to poor clinical outcomes in patients.^6^ Drug metabolism, together with epithelial‐mesenchymal transition (EMT) and TME, appear to play a crucial role in PC chemotherapeutic resistance.^7^ Here, we have concluded the principal mechanisms of gemcitabine resistance in PC (Figure 1). Cellular uptake of gemcitabine (GEM) is mainly mediated by sodium‐dependent and sodium‐independent transporters.^8^ Decreased expression of hENT1 results in GEM resistance in PC.^7^ Overexpression of ABCC5, an ATP binding cassette (ABC) transporter, causes 5‐FU or GEM resistance in PC.^9^ Cytidine deaminase (CDA) inactivates intracellular GEM and reduces the sensitivity of PC cells to GEM.^10^, ^11^ Besides, glutathione peroxidase‐1 (GPx1) sensitizes pancreatic ductal adenocarcinoma (PDAC) cells to GEM and suppresses EMT by inhibiting Akt/GSK3β/Snail signaling pathways.^12^ GPx1‐silenced PDAC cells are related to the increased resistance of cancer cells to GEM.^12^ Various signaling pathways are involved in the capability of tumor cells to develop resistance to chemotherapies. Aside from multiple non‐neoplastic cells such as cancer‐associated fibroblasts, immune cells, and neurons, the extracellular matrix (ECM) components such as collagen and hyaluronic acid are contained in TME.^13^ Neoplastic cells and stromal cells are bidirectionally linked through dynamical feedback.^14^ Reciprocal signaling interactions between cancer cells and stromal cells contribute to the development of malignant stages and aggressive phenotypes of cancer.^14^

Exosomes are extracellular vesicles that are synthesized by prokaryotic and eukaryotic cells.^15^ Exosomes carrying proteins, lipids, DNA, and RNA are secreted into the extracellular space and absorbed by target cells.^16^ Exosomes are present in various body fluids such as blood, urine, saliva, malignant ascites, amniotic fluid, bronchoalveolar lavage fluid, malignant effusions of ascites, breast milk, and synovial fluid.^15^, ^17^ Exosomes can be transferred among different cells and among body fluids, which physiologically and pathologically modulate biological responses.^18^ Accumulating evidence has proved that exosomes are involved in many processes of PC, such as metastasis, cell proliferation, EMT, angiogenesis, and TME.^19^ In particular, the in vitro, pre‐clinical in vivo and patients' data have shown that extracellular vesicles are related to PC.^20^, ^21^ Exosomes are reported to regulate PC drug resistance through triggering drug efflux, inducing anti‐apoptotic activity and epithelial to mesenchymal transition (EMT), mediating inactivation of chemotherapeutic drugs, and so on.^22^, ^23^, ^24^, ^25^ In this review, we aim to discuss the current knowledge on the role of exosomes in PC chemoresistance and propose possible therapeutic interventions to overcome it.

## 2 EXOSOME BIOGENESIS AND CELLULAR ENTRY

As a type of single‐membrane vesicles commonly isolated by ultracentrifugation in vitro, exosomes are secreted after the fusion of multivesicular endosomes with the cell surface *in vivo*.^15^, ^26^ Budding, invagination, multivesicular bodies (MVBs) formation, and secretion are the four steps in the production of exosomes.^27^ The invagination of the plasma membrane forms a cup‐shaped structure and leads to the formation of an early‐sorting endosome (ESE) as a type of budding.^15^, ^27^ ESEs receive endocytic cargo in a variety of ways, including the clathrin‐mediated pathway.^28^ ESEs can either return the cargo to the plasma membrane as “recycling endosomes” or mature into late‐sorting endosomes (LSEs).^29^ LSEs then transform into MVBs.^15^ MVBs form by the inward invagination of the endosomal limiting membrane, which results in MVBs containing multiple intraluminal vesicles (ILVs).^15^ MVBs are either degraded by fusing with lysosomes or secreted to release the contained ILVs as exosomes with the help of RABs, actin, and SNARE proteins.^30^ The main molecular mechanisms for the formation of exosomes are the endosomal sorting complexes required for transport (ESCRT) and non‐ESCRT.^30^, ^31^ The multiprotein complex ESCRT dominates the membrane invagination of intraluminal vesicles into the multivesicular bodies and causes the release of exosomes into the extracellular space.^32^

Exosomes can be taken up via phagocytosis, micropinocytosis, and endocytosis, as well as by fusing with the plasma membrane.^33^, ^34^ The mechanisms of exosome uptake and cargo delivery into the cytoplasm of acceptor cells, on the other hand, are still poorly understood. Some studies have shown that exosomes can be internalized in a cell type‐specific manner that is dependent on cell or tissue recognition of exosomal surface molecules.^27^ For example, oligodendrocyte‐derived exosomes are specifically and efficiently taken up by microglia.^35^ However, very little exosome uptake was observed in astrocytes or neurons in the cerebral cortex or hippocampus.^36^ It is both difficult and fascinating to gain a more comprehensive understanding of exosomes.

## 3 BIOLOGICAL ACTIVITIES AND THERAPEUTIC APPLICATIONS OF EXOSOMES

Exosomes are involved in multiple physiological processes, such as antigen presentation, inflammation, coagulation, cellular homeostasis, apoptosis, intercellular signaling, and pathological states, such as infections, cancer, neurodegenerative disease, pregnancy, cardiovascular diseases, immunoregulation, autoimmune.^15^, ^30^, ^37^, ^38^ Considering the biological features of those vesicles, research has extensively explored the relationship between the exosomes with both diagnosis and prognosis in human disease. Lipoprotein receptor‐related protein 6, heat‐shock factor‐1, and repressor element 1‐silencing transcription factor in exosomes show good diagnostic value in patients with Alzheimer's disease.^39^ Exosomal miR‐1 and miR‐133a levels increased earlier than serum creatine phosphokinase and cardiac troponin T in infarcted regions of the heart, indicating a promising diagnostic method for ischemic heart disease.^40^, ^41^ MiR‐375‐3p, let‐7c‐5p, MiR‐362‐3p, miR‐877‐3p, miR‐150‐5p, and miR‐15a‐5p were upregulated in the urine and serum of diabetics, exosomal RNA may therefore be novel biomarkers for detecting diabetes mellitus.^42^, ^43^, ^44^

Based on the role of exosomes as natural carriers of proteins, metabolites, and nucleic acids, much attention has been paid to the field of exosome‐based drug delivery. Exosomal miR‐146b decreases EGFR and NF‐κB protein in glioma cells and therefore reduced glioma growth in vitro.^45^ The siCPT1A loading iRGD‐engineered exosomes not only showed efficient tumor targeting but also reversed oxaliplatin resistance in colon cancer, which expanded the application of siRNA‐based anti‐tumor therapy and provide a new strategy for treating oxaliplatin‐resistant colon cancer.^46^ Through the fusion of the gene‐engineered exosomes with thermosensitive liposomes, therapeutic nanovesicles hGLV was formed.^47^ ICG and R837 co‐encapsulated hGLV provided a nano‐drug delivery system in cancer treatment by combining photothermal therapy with immunotherapy.^47^ These studies emphasize the biological importance of exosomes.

## 4 FUNCTIONS OF EXOSOMES ON PC CHEMORESISTANCE

Chemoresistance becomes a major clinical challenge in anti‐cancer therapies as chemoresistant cancer cells usually present more aggressive biological behavior.^48^ In vitro, pre‐clinical in vivo, and patients' data have already shown that there is an interaction between extracellular vesicles and drug resistance in lung cancer, ovarian cancer, hematological malignancies, gastric cancer, kidney cancer, osteosarcoma, and PC.^23^, ^49^, ^50^, ^51^, ^52^, ^53^, ^54^ Chemoresistance has partly contributed to the dismal prognosis of PC.^55^ As a kind of extracellular vesicles, PC‐derived exosomes play significant roles in drug resistance of cancer.^56^, ^57^ Thus, exploring the relation between exosomes and chemoresistance may be conducive to understanding molecular mechanisms and taking effective measures to reduce the development of drug resistance. We concluded the underlying mechanisms of exosome in drug resistance of PC (Table 1).

**TABLE 1:** The exosomal factors involved in the chemoresistance of pancreatic cancer

Donor cells	Exosomal contents	Recipient cells	Functions	Mechanisms	Refs.
PANC‐1	EphA2	MIA PaCa‐2 and BxPC‐3	Induce chemoresistance of gemcitabine	Not yet clear	^110^
Panc1 and MiaPaCa2	MiR‐155	–	Promote gemcitabine resistance in vivo	Result in anti‐apoptotic activity by targeting TP53INP1	^23^
CAFs	Snail mRNA	Pancreatic cancer epithelial cells	Induce chemoresistance of gemcitabine in vitro	Not yet clear	^24^
BxR‐CSCs	MiR‐210	BxS and PANC‐1	Induce chemoresistance of gemcitabine	Trigger the mTOR signaling pathway.	^86^
TAM	MiR‐365	PDAC cells	Induce chemoresistance of gemcitabine in vitro	Upregulate the triphospho‐nucleotide pool in cancer cells and induce the enzyme cytidine deaminase	^25^
Gemcitabine‐treated PC cells	*SOD2* and *CAT* transcripts	–	Induce chemoresistance of gemcitabine in vitro	Suppress basal and gemcitabine‐induced ROS production	^102^
Gemcitabine‐treated PC cells	MiR‐155	–	Induce chemoresistance of gemcitabine in vitro	Downregulate *DCK*	^102^
CAFs	MiR‐106b	–	Promote gemcitabine resistance	Target TP53INP1	^76^
GIPC‐depleted AsPC‐1 and PANC‐1 cells	ABCG2	–	Induce chemoresistance of gemcitabine in vitro	Serve as drug efflux transporter protein	^22^
BxPC‐3‐Gem cells	MMP14	BxPC‐3 and Mia‐PaCa‐2cells	Promote gemcitabine resistance	Increased cancer stemness and invasion properties	^92^

Abbreviations: EphA2, Ephrin type‐A receptor 2; TP53INP1, tumor protein 53‐induced nuclear protein 1; CAFs, cancer‐associated fibroblasts; CSCs, cancer stem cells; mTOR, mammalian target of rapamycin; TAM, tumor‐associated macrophages; PDAC, pancreatic ductal adenocarcinoma; PC, pancreatic cancer; ABCG2, The ATP‐binding cassette (ABC) superfamily G member 2; MMP14, matrix metalloproteinase 14.

### 4.1 Exosomes‐triggered drug efflux regulate drug resistance

The expression of drug efflux pumps on the membranes of tumor cells is a major cause of drug resistance.^58^ At least 20 ABC transporters are responsible for the efflux of anticancer agents.^59^ These transporters or drug efflux pumps within the human body include proteins of the ATP‐Binding Cassette (ABC) superfamily such as P‐glycoprotein (P‐gp, MDR1, or ABCB1), multidrug resistance‐associated protein 1 (MRP1 or ABCC1), and mitoxantrone resistance protein (MXR, ABCG2, or the breast cancer resistance protein).^58^ ABCC1 is characterized by tissue‐specific expression in various cancer types, including lung cancer, breast cancer, liver cancer, brain cancer, renal cancer, and so on.^60^ Wang et al. illustrated that human breast cancer MCF‐7/ADR cells derived‐exosome carried MDR‐1 mRNA and its product P‐gp that could be transferred between cells and move away intracellular antitumor agents to facilitate the dissemination of drug resistance via horizontal transfer.^61^ High‐level expression of both ABCB1 and ABCG2 in hepatocellular carcinoma and kidney cancer makes these types of tumors refractory to chemotherapy.^62^Increased autophagy and secretion of exosomes were identified in PC cells after the depletion of GAIP interacting protein C‐terminus (GIPC).^22^ The depletion of GIPC and overexpression of the drug resistance gene *ABCG2* in exosomes sensitized PANC‐1 cells.^22^ The above results suggest that the involvement of GIPC promotes the formation of more resistance phenotypes of PC by regulating *ABCG2*.^22^ These findings can be further explored as new therapeutic methods to overcome drug resistance in cancers such as PC.

### 4.2 Exosomes induce anti‐apoptotic activity in drug resistance

Cell apoptosis, in contrast to necrosis, is characterized by cell shrinkage, nuclear condensation and fragmentation, cleavage of chromosomal DNA, and packaging into apoptotic bodies without the ultimate breakdown of the plasma membrane.^63^, ^64^ Apoptosis refers to the activation of an intrinsic suicide program and systematical destruction of cells. Apart from functioning in physiological processes, apoptosis is operational during diverse pathological processes such as tumor growth, immune response, and neurodegeneration.^65^ The intrinsic and extrinsic pathways of cell apoptosis are shown to be triggered by cellular stress, DNA damage, and immune surveillance mechanisms.^66^ Multiple kinds of drugs that target the apoptotic pathway have been proven to be effective for cancer treatment, indicating that apoptotic pathways in tumor cells are potent anti‐cancer targets.^66^ These targeted agents include inhibitors of growth factor signaling pathways, kinases, mammalian target of rapamycin(mTOR), proteasomes, and histone deacetylases.^66^ Weakening GEM‐induced apoptosis is one of the latent mechanisms causing drug resistance in PDAC.^67^

#### 4.2.1 Exosomes induce apoptosis of lymphoid cells

Immunosuppressive TME remains one of the main unfavorable factors for the development and drug resistance of PC.^68^, ^69^ Exosomes derived from cancer cells inhibit the immune response of the body to tumor cells by inducing apoptosis of lymphoid cells.^70^ A previous study revealed that PC‐derived exosomes induced ER stress‐mediated apoptosis of T lymphocytes via p38 MAPK, engendering immunosuppression and the reduced effectiveness of immunotherapy.^71^ Although detailed interaction mechanisms between tumors and lymphocytes are complex and less known, the available information on the immune microenvironment prompts us to explore further.

#### 4.2.2 Exosomes induce anti‐apoptotic activity of PC cells

Tumor protein 53‐induced nuclear protein 1 (TP53INP1), a proapoptotic stress‐induced *p53* target gene, can be repressed by the oncogenic miRNA.^72^, ^73^ TP53INP1 acted as a stress‐induced protein that promoted apoptosis in response to DNA damage and *p53* phosphorylation at Ser‐46.^74^, ^75^ Recent studies demonstrated that TP53INP1 was associated with chemoresistance of breast cancer by potentiating drug‐induced apoptosis in cancer cells.^73^ Expression of miR‐155 in PDAC cells increased with long‐term exposure to GEM.^23^ The increase of miR‐155 not only induced chemoresistance via enhancing anti‐apoptotic activity but also promoted exosome secretion to deliver miR‐155 into other PDAC cells.^23^ Nevertheless, the underlying mechanisms of how miR‐155 effectively promotes exosome secretion in PC cells through such a positive feedback process remain unknown. Similarly, exosomal miR‐106b derived from cancer‐associated fibroblasts served a crucial role in GEM resistance by targeting TP53INP1 in PC.^76^ B‐cell translocation gene 2 (*BTG2*) is involved in numerous important biological processes in cancer cells acting as a tumor suppressor.^77^ MiR‐27a silencing attenuated proliferation and invasion of PC cells by promoting apoptosis through the increased expression of *BTG2*.^78^ In addition, PC cells‐derived exosomes carrying miR‐27a promoted human microvascular endothelial cells (HMVEC) angiogenesis via *BTG2* in PC.^78^ Hence exosome‐derived miR‐27a may be a potential target for PC treatment.

The mammalian target of rapamycin (mTOR) is a serine/threonine kinase regulating numerous fundamental cellular processes, which include protein synthesis, metabolism, growth, autophagy, and others.^79^, ^80^ The kinase mTOR functions as a master regulator of the PI3K‐Akt–mTOR pathway which is considered the most deregulated signaling pathway in cancer.^81^ The hyperactivation of either upstream members of mTOR such as PIK3CA, RAS (H, K, and NRAS), and Akt, or downstream effectors of p70S6K, 4EBP1, and eIF4, resulted in the deregulation of the mTOR signaling pathway.^81^ The mTOR signaling is a major compensatory pathway conferring drug resistance to anti‐tumor agents in an autonomous or non‐cell‐autonomous manner.^82^ Downregulation of mTOR has widely been found in multiple human cancers, such as breast, prostate, lung, liver, and renal carcinomas.^83^ MLN0128 (also called INK128, sapanisertib, TAK‐228) is a pan‐mTOR inhibitor that has potent anti‐tumor effects in PIK3CA‐mutant colorectal cancer and CD44‐high HCC xenografts.^84^, ^85^ Moreover, ATP‐competitive mTORC1/2 inhibitors, such as AZD2014 (vistusertib) and its analog AZD8055, are highly effective in treating estrogen receptor (ER)‐positive breast cancer.^83^ BxR‐CSC‐derived exosomes inhibited GEM‐induced cell cycle arrest and antagonized GEM‐induced apoptosis, thus inducing GEM resistance in PC.^86^ Notably, the above characteristic of chemotherapy drug resistance was related to the horizontal transfer of miR‐210 from GEM‐resistant PC cell‐derived exosomes.^86^ The discovery of elevated phosphorylation of mTOR and its downstream target S6K1 demonstrated that miR‐210 carried by BxR‐CSCs/Exo mediated the transfer of the resistance phenotype to PC cells by triggering the mTOR signaling pathway.^86^ These studies provide novel insights to develop potential strategies for the treatment of PC via inhibiting the anti‐apoptotic activity of PC cells.

### 4.3 Exosomes engender PC chemoresistance by EMT

As the name suggests, epithelial to mesenchymal transition (EMT) refers to the transdifferentiation of epithelial cells into motile mesenchymal cells.^87^ EMT is involved in various biological processes, including development, wound healing, fibrosis, and cancer progression.^88^ As an EMT transcription factor, SNAIL protein contributes to the repression of the epithelial phenotype and the activation of the mesenchymal phenotype.^88^ EMT program plays a role in suppressing drug transporters and concentrating proteins and therefore protecting EMT+ cells from antineoplastic drugs such as GEM.^89^ Cancer‐associated fibroblasts (CAFs), the most abundant cells in TME, are involved in several cancer progressions including tumor relapse and therapeutic resistance.^90^ There is a close correspondence between the activation of the EMT program and the entrance of tumor cells into the CSC state.^91^ Snail mRNA levels were highly increased in exosomes which were heavily secreted by CAFs during GEM treatment, promoting proliferation and chemoresistance of PDAC epithelial cells.^24^

Matrix metalloproteinase 14 (MMP14) is a crucial molecule in the intercellular communication process. MMP14 promoted gemcitabine resistance in sensitive PDAC cells through exosome transmission.^92^ Exosome‐transferred MMP14 boosted the stability of CD44 protein in recipient cells, according to a protein stability experiment.^92^ CD44, in conjunction with other cell surface markers, has been widely used to characterize CSCs in a variety of solid tumors.^93^ CD44 can regulate TGF‐mediated EMT to maintain CSCs and protect CSCs from reactive oxygen species (ROS).^94^, ^95^ As a result, MMP14 is a critical player in the exosome‐mediated transmission of chemoresistance.

### 4.4 Exosomes mediate inactivation of chemotherapeutic drugs

Besides the main metabolic organs such as the liver and kidney, the drug metabolism processes within tumors are also closely associated with the effectiveness and toxicity of chemotherapeutic drugs.^96^, ^97^ Abnormal expressions and activity of metabolic enzymes have been found in liver cancer, breast cancer, gastrointestinal cancer, lung cancer, and PC.^98^ Drug‐metabolizing enzymes (DMEs) trigger drug resistance by inactivation and detoxification of chemotherapeutic agents within tumor tissue or metabolic organs.^99^ UDP glucuronosyltransferases (UGTs) are phase II drug‐metabolizing enzymes.^100^ Lower amounts of UGT2B4 and UGT2B7 isoforms were expressed in breast or pancreatic cancer than that in normal tissues, which demonstrated that the reintroduction of UGTs has the potential to reduce lipids needed for rapid cancer cell division and further trigger cell death.^101^ It has recently been shown that macrophages‐derived exosomes mediated the transfer of miR‐365 to PDAC cells followed by modulating GEM metabolism.^25^ MiR‐365 upregulated pyrimidine metabolism and increased NTP levels of cancer cells.^25^ CDA, which was a kind of enzyme responsible for GEM inactivation in humans, was then upregulated in response to NTP.^25^ This suggests miR‐365 in macrophages‐derived exosomes is a resistance factor with important clinical implications in PC patients. A related study showed that conditioned media (CM) of GEM‐treated PC cells (Gem‐CM) and its EV fraction (Gem‐EV) conferred chemoresistance to PC cells.^102^ The level of superoxide dismutase 2 (SOD2) and catalase (CAT; ROS‐detoxifying enzymes) in PC cells increased through exosome‐mediated lateral transfer of their transcripts.^102^ Gem‐Exo‐mediated delivery of miR‐155 causes downregulation of GEM‐metabolizing enzyme DCK in PC cells by directly targeting its 3′‐UTR.^102^ All these processes are known to promote acquired GEM resistance of PC cells. Nevertheless, more biological mechanisms underlying exosomes and the metabolism of drugs in organisms demand further exploration.

### 4.5 Other mechanisms remain to be elaborated

Ephrin type‐A receptor 2(EphA2) is expressed more abundantly in tumor tissues compared to most normal tissue.^103^, ^104^ Ephrin receptors are receptor tyrosine kinases (RTKs) and have been attracting more and more attention because of their capacities to modulate processes controlling tumor migration and invasion.^104^ Angiogenesis of tumor cells was promoted by the migration and sprouting of EphA2‐specific endothelial cells and the increased expression of vascular endothelial growth factor.^104^ EphA2 expression in cancer cells caused immunosuppression in the TME and therefore conferred resistance to combination immunotherapy through EPHA2/TGF‐β/SMAD axis–dependent activation of prostaglandin‐endoperoxide synthase 2.^105^ Moreover, EphA2 induces chemotherapy resistance through various signaling pathways in several types of tumors, including gastric cancer, high‐grade serous ovarian cancer, clear cell renal cell carcinoma, melanoma, and PC.^106^, ^107^, ^108^, ^109^ Besides acting as a biomarker‐based diagnostic method using a combination of Ephrin type‐A receptor 2 in exosomes (Exo‐EphA2), CA 199, and CA 242, Exo‐EphA2 can confer resistance of GEM‐sensitive PC cells to GEM.^110^, ^111^ The dose‐dependent increase in EphA2 expression was found in MIA PaCa‐2 and BxPC‐3 (GEM‐sensitive) cells when incubating these GEM‐sensitive cells with PANC‐1(GEM‐resistant) exosomes.^110^ PANC‐1 cells expressing EphA2‐shRNA‐1 indicated a ~80% decrease in EphA2 expression and a ~25% decrease in chemoresistance to GEM.^110^ Conjugating GEM with artificially designed EphA2 binding ligands revealed excellent therapeutic efficacy in the animal models of PC.^112^ Thus, exosome‐mediated EphA2 expression in cancer cells plays an essential part in tumor drug resistance.

## 5 CONCLUSION

Considering the role of exosomes in the transfer of chemoresistance, inhibiting exosomes biogenesis or release from donor cells, restricting the export of drug‐resistant cargos from exosomes, and preventing exosomes from their interaction with recipient cells may be potential and beneficial strategies in overcoming the drug resistance in PC. Beyond these therapeutic interventions, removing or destroying malicious exosomes that existed in the TME or circulation by physical or chemical methods may be effective. Despite a part of regulatory sites have been found during the development of exosome‐mediated drug resistance in PC, exploring effective PC treatment methods that would be able to interfere with these regulatory sites without affecting normal cells remains a dilemma.

Exosomes may be thought of as a potential biomarker for predicting drug resistance and prognosis in PC. Furthermore, the combination of human serum exosomes and existing markers such as CA199 may improve the diagnosis rate of early PC. Since exosomes are involved in multiple pathophysiological processes, loading therapeutic agents such as tumor‐suppressing proteins, nucleic acid, and targeted drugs into exosomes may help to develop precision medicine. Exosomes are tentatively shown to be a promising target to reverse exosome‐mediated drug resistance. However, the complex biological behavior of exosomes in cancer is still not fully elucidated. Further preclinical and multicenter clinical validation studies are still needed.

## AUTHOR CONTRIBUTIONS

Yubin Pan participated in the design of the study and wrote the manuscript. Honglin Tang wrote the manuscript and critically reviewed the manuscript. Qijun Li and Guangpeng Chen critically reviewed the manuscript. Da Li conceived the study and critically revised the manuscript. All the authors read and approved the final form of the manuscript.

## CONFLICT OF INTEREST

The authors declare that there is no conflict of interest.

## ETHICAL STATEMENT

This is a review article and the need for ethics approval and consent was waived.

## Data Availability

Data sharing is not applicable to this article as no new data were created or analyzed in this study.
